# The effect of using dental loupes on the accuracy of aligner attachments and flash adhesive area after bonding

**DOI:** 10.1590/2177-6709.31.2.e2625190.oar

**Published:** 2026-05-18

**Authors:** Servet ERBAŞ, Ezgi ATIK

**Affiliations:** 1 Private clinician (Ankara, Turkey).; 2 University of Hacettepe, Dental School, Department of Orthodontics (Ankara, Turkey).

**Keywords:** Dental loupe, Clear aligner attachment, Orthodontic bonding, Accuracy, Flash adhesive, Lupa odontológica, *Attachment* de alinhador estético, Colagem ortodôntica, Acurácia, Excesso de adesivo

## Abstract

**Objective:**

This study aimed to assess whether the use of dental loupes improves the accuracy of aligner attachments and reduces the amount of composite flash after bonding.

**Methods:**

Six epoxy resin-printed models (n=14 teeth per model) were created to produce upper arches. All attachments were bonded with G-ænial Posterior composite using the same aligner template, and the protocols differed regarding the use of a dental loupe. The attachments were bonded using the dental magnifying loupe at 2.5X magnification in the “with loupe” Group 1 (n=42 teeth), and with the naked eye in the “without loupe” Group 2 (n=42 teeth). All bonded attachments were scanned and compared to those of a reference model using Geomagic Control X software to assess accuracy, with a focus on the highest deviations (defects and excesses) between the reference attachment and the results from different bonding protocols. The amounts of flash adhesive were also measured. Independent Samples T-test and Mann-Whitney U test were used. The significance level was set at 0.05.

**Results:**

The maximum deviation in excess was 0.46 mm (0.2-1.21) in Group 2, which was higher than the value in Group 1 (0.38 mm, 0.18-0.79) (p=0.048). Regarding the flash adhesive area, the “without loupe” group showed a significantly higher value (42.01±10.32 mm²) compared to the “with loupe” group (31.79±5.38 mm²) (p<0.001).

**Conclusions:**

Using a magnifying dental loupe revealed significantly lower values in both the flash adhesive area around the attachment and the maximum vertical excess on the attachment surface. Clinicians are advised to use dental loupes during bonding attachments.

## RESUMO

**Objetivo:**

Este estudo teve como objetivo avaliar se o uso de lupas odontológicas melhora a precisão na colagem de *attachments* em alinhadores e reduz a quantidade de excesso de resina após a colagem.

**Métodos:**

Seis modelos impressos em resina epóxi (n=14 dentes por modelo) foram criados para produzir arcadas superiores. Todos os *attachments* foram colados com o composto G-ænial Posterior utilizando o mesmo *template* de alinhador, e os protocolos diferiram quanto ao uso de lupa odontológica. Os *attachments* foram colados utilizando uma lupa de magnificação odontológica de 2,5X no Grupo 1 “com lupa” (n=42 dentes), e a olho nu no Grupo 2 “sem lupa” (n=42 dentes). Todos os *attachments* colados foram escaneados e comparados aos de um modelo de referência, utilizando o *software* Geomagic Control X para avaliar a acurácia, com foco nos maiores desvios (defeitos e excessos) entre o *attachment* de referência e os resultados dos diferentes protocolos de colagem. As quantidades de excesso de adesivo também foram medidas. Foram utilizados o teste t para amostras independentes e o teste U de Mann-Whitney. O nível de significância foi definido em p < 0,05.

**Resultados:**

O desvio máximo em excesso foi de 0,46 mm (0,2-1,21) no Grupo 2, o qual foi superior ao valor do Grupo 1 (0,38 mm, 0,18-0,79) (p=0,048). Em relação à área de excesso de adesivo, o grupo “sem lupa” apresentou um valor significativamente maior (42,01±10,32 mm^2^), em comparação ao grupo “com lupa” (31,79±5,38 mm^2^) (p<0,001).

**Conclusões:**

O uso de uma lupa odontológica revelou valores significativamente menores tanto na área de excesso de adesivo ao redor do *attachment* quanto no excesso vertical máximo na superfície do *attachment*. Recomenda-se que os clínicos utilizem lupas odontológicas durante a colagem de *attachments*.

## INTRODUCTION

In fixed orthodontic treatment, flash adhesive may be present around the bracket-enamel interface during bonding. Clinicians should remove any excess resin after placing the bracket, before curing the adhesive. If the adhesive remnant on the tooth surface after bracket bonding is not thoroughly removed, it can create a rough surface that promotes microbial growth and colonization.^1^ ^,^ ^2^ This situation could result in a higher occurrence of white spot lesions and demineralization.^3^ ^,^ ^4^ Resin removal presents a challenge for clinicians because it is difficult to differentiate adhesive remnants from the tooth structure, mainly due to recent aesthetic advancements in restorative materials.^5^ Indeed, over time, adhesive remnant materials undergo aging processes that can lead to discoloration and subsequently impact their aesthetic results.^6^ Therefore, preventing adhesive overflow during the initial stage of the orthodontic bonding procedure should be a primary goal. 

Studies have shown that complex tooth movements -such as incisor torque, rotation, and molar distalization- can be effectively achieved in clear aligner therapy through the use of auxiliaries like composite attachments.^7^ ^,^ ^8^ Although using attachments provides several benefits, the bonding process may inevitably result in excess flash, either due to the flowable characteristics of the composite resin or the over-application of packable composite materials. Several studies have investigated the influence of bonding protocols and composite types on the accuracy of aligner attachment shapes and the amount of flash generated, yielding significant results.^9^ ^-^ ^13^

Dental loupes have been used in clinical dentistry for many years, offering various magnification levels, depths of field, weights, and built-in lighting systems to support precise and detailed work visualization.^14^ ^,^ ^15^ This popular tool enhances visibility, encourages better posture, and reduces strain for dental professionals.^16^ ^,^ ^17^

The use of magnification tools in Orthodontics is primarily advised to enhance the accuracy of bracket placement, lingual appliance positioning, and removal of flash adhesive during debonding. Most studies conducted^18^ ^-^ ^21^ have focused on these aspects. A systematic review presenting updated scientific evidence supporting the use of dental loupes emphasizes the need for more clinical trials to investigate long-term outcomes of different procedures.^22^ One recent study observed that the amount of adhesive remnants was influenced by the use of dental loupes.^23^

To the best of our knowledge, no study has assessed the effectiveness of dental loupes on aligner attachment accuracy and the amount of flash adhesive after bonding aligner attachments to tooth surfaces. In this regard, the present *in vitro* study aimed to evaluate both the accuracy of attachment positioning and flash adhesive area around attachments with and without the use of dental loupes. The null hypothesis of the study is that there would be difference in bonding accuracy and flash area for aligner attachments between using loupes and the direct eye vision method (without loupes). 

## MATERIAL AND METHODS

### SAMPLE PREPARATION

As the present *in vitro* study was conducted using epoxy resin models without involving human or animal subjects, ethical approval was deemed unnecessary. Sample size calculation was determined using G*Power software (G*Power v.3.0.10; Franz Faul, Universität Kiel, Germany). According to the study conducted by D’Antò et al.^9^, a statistically significant difference was found in the amount of flash adhesive around attachments between the two types of composite resins (resin 1: 0.340 ± 0.100 mm; resin 2: 0.433 ± 0.121 mm). Based on these values, assuming an effect size of 0.837, at least 32 specimens per group were needed to achieve 95% power with a significance level of 0.05. Due to the potential for sample loss, 42 specimens were assigned to each group.

**Figure 1:**
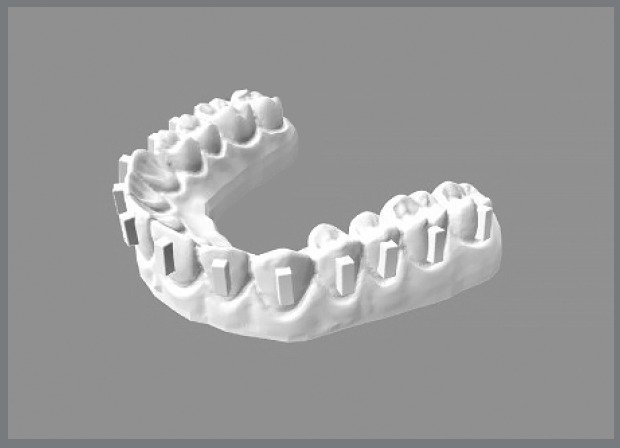
Virtual (reference) model showing the vertical conventional rectangular attachments with dimensions of 5 mm × 2 mm × 1.25 mm.

Six epoxy resin-printed maxillary models (n=14 teeth per model, extending from the right second molar to the left second molar) were created to simulate realistic upper dental arches. These models were scanned using an Itero 5 intraoral scanner (Align Technology). The vertical conventional rectangular attachments, measuring 5 mm × 2 mm × 1.25 mm, were digitally positioned at the middle third of each tooth using OnyxCeph3™ software (Fig. 1). Customized attachment templates were then created for each group to streamline the bonding process.

### BONDING PROCEDURE OF COMPOSITE ATTACHMENTS

All attachments were bonded with G-ænial posterior packable composite resin (GC Corporation, Tokyo, Japan). The bonding process was performed without using etching or adhesive primer. A metal packing hand instrument was used to carefully condense the composite material into the reservoir of the attachment template, promoting optimal adaptation. The bonding procedure for three epoxy resin dental models (Group 1: with loupe, n=42 teeth) was performed using a dental loupe at 2.5X magnification. In comparison, the remaining three dental models (Group 2: without loupe, n=42 teeth) were bonded with the naked eye, without a dental loupe. The attachment templates were fully seated onto the study arch models with gentle occlusal and labial pressure. Composite attachments were light-cured for 6 seconds (3 seconds mesial and 3 seconds distal) using the VALO Ortho LED curing unit (Ultradent Products, South Jordan, Utah) at an intensity of 1200 mW/cm². Only one experienced operator performed the bonding procedure on all teeth, from the right second molar to the left second molar, for each model, to ensure procedural standardization. The dental loupe used (Ymarda Dental Loupe CH250-RTPSL-2.5, Silver) had a 2.5X magnification and a working distance of 350 mm in Group 1. In both groups, a conventional dental reflector was used to illuminate specimens during bonding procedures. The reflector’s position was previously standardized for distance and angle.

### EVALUATION OF ATTACHMENT ACCURACY AND FLASH ADHESIVE AMOUNT WITH SUPERIMPOSITION TECHNIQUE

**Figure 2:**
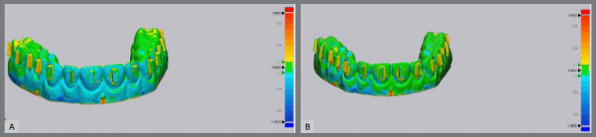
A) Examples of superimposition of the models for Group1 (with dental loupe) and B) for Group 2 (naked eye without dental loupe)

**Figure 3:**
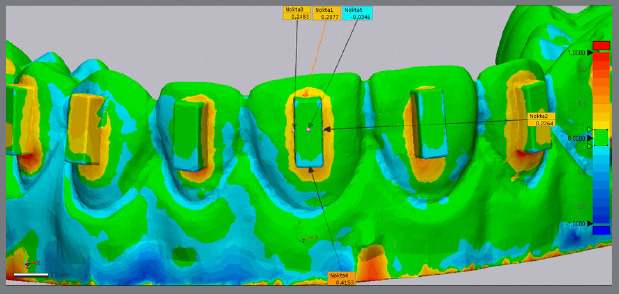
The superimposition accuracy between the reference values examined with the “3D Compare” command.

**Figure 4:**
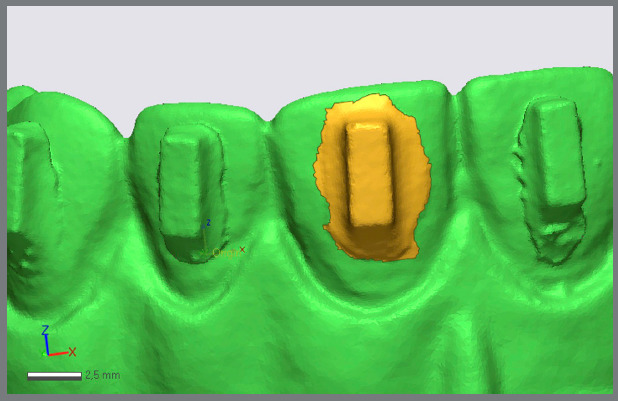
The measurement of flash composite surface area (mm²) around the attachment using the software’s “Area Calculate” function.

After finishing the bonding process, the STL (stereolithography) images of the six dental models were created using the Itero 5 intraoral digital scanner (Align Technology). The comparison of the attachments on the experimental models (realized) with those of the virtual models (reference model) was performed using a reverse engineering analysis 3D Geomagic Control X software (3DS Systems, version 2015, Rock Hill South Carolina, USA) by a qualified mechanical engineer (Fig. 2). To minimize potential bias, the software operator responsible for the superimpositions and the calculations of excess, deficit, and surface area was blinded to the study purpose, methodology, and group allocation. To perform “Best-Fit Alignment”, the reference model was segmented using the “Resegment” tool command. The occlusal surfaces of the teeth were defined as individual segments. The accuracy of superimposition between the reference values was assessed using the “3D Compare” command (Fig. 3). A color-coded map highlighting the “defect” and “excess” areas for the attachment shape was used to illustrate the morphological differences between the reference and experimental models. By utilizing the methodology from a previous study^12^, the maximum deviation was set to ±1 mm, and the color coding was defined as follows: green = perfectly matched surface (error ±0.1 mm); yellow = excess areas (error between +0.1 and +0.8 mm); and blue = deficiency areas (error between -0.1 mm and -0.8 mm). The engineer using the software identified the maximum deficiency and excess measurements. To quantify the surface area (mm²) of excess composite around the attachment, the flash material was segmented as a single unit and analyzed using the software’s “Area Calculate” function (Fig. 4).

### STATISTICAL ANALYSIS

The data obtained in the study were analyzed using the IBM SPSS (Statistical Package for the Social Sciences) version 25.0 software. The descriptive statistics were presented as numbers, percentages, means, standard deviations, medians, minimum values, and maximum values.

The assumption of normality was tested with the Shapiro-Wilk test, and the homogeneity of variances was evaluated using Levene’s test. In cases where the assumption of normality was met, the Independent Sample T-test was used to compare two independent groups (flash adhesive area measurement); otherwise, the Mann-Whitney U-test was employed when the normality assumption was not met (accuracy measurements). Variables with normal distribution were reported as mean ± standard deviation, whereas non-normally distributed variables were presented as median and interquartile range. The significance level was set at p<0.05.

## RESULTS

**Figure 5:**
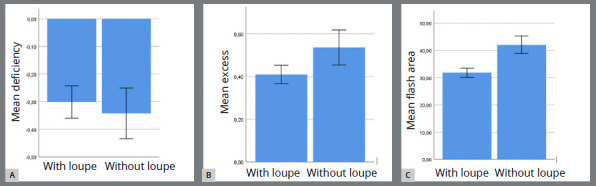
Histogram graph of **A)** “defect” measurement of attachment accuracy, **B)** “excess measurement of attachment accuracy, **C)** “flash area” measurement between different bonding groups.

There was no significant difference between the groups in the mean “defect” measurement of placement accuracy, as shown in Table 1 (p=0.934) (Fig. 5A).

**Table 1:** Comparison of the attachment accuracy and flash adhesive area measurements between different bonding groups.

Variables	Group 1 (n=42) With loupe		Group 2 (n=42) Without loupe		Test Statistics	p-value
	Median (Min./Max.)	Mean±SD	Median (Min./Max.)	Mean±SD		
“Defect” accuracy measurement (mm)	-0.25 -0.64/-0.13	-0.30±0.14	-0.26 -1/-0.12	-0.34±0.24	-0.083^a^	0.934
“Excess” accuracy measurement (mm)	0.38 0.18/0.79	0.41±0.14	0.46 0.2/1.21	0.54±0.26	-1.977^a^	0.048*
Flash adhesive area (mm^2^)	30.68 23.02/42.77	31.79±5.38	42.32 24.57/67.5	42.01±10.32	-5.696^b^	<0.001*

^a^ Mann-Whitney U-test was applied. ^b^ Independent Sample T-test was applied. Min.=Minimum; Max. =Maximum; SD: Standard deviation. *p<0.05 statistically significant.

The median “excess” measurement accuracy of the “with loupe” group (0.38 mm [0.18-0.79]) was significantly lower than that of the “without loupe” group (0.46 mm [0.2-1.21]) (p=0.048) (Table 1 and Fig. 5B).

Considering the mean values of the overflowing flash adhesive area, a significant difference was found between the “with loupe” (31.79±5.38 mm²) and “without loupe” group measurements (42.01±10.32 mm²). The “without loupe” group showed significantly higher values than the “with loupe” group (p<0.001) (Table 1 and Fig. 5C).

## DISCUSSION

In fixed bracket systems, excess composite around the bracket can be removed before light curing; however, this is not easily achievable in aligner treatments. During aligner attachment bonding, if the clinician places an excessive amount of adhesive into the attachment reservoirs of the template, the risk of flash formation increases. Therefore, it is crucial to apply an adequate amount of adhesive into the reservoir spaces of the template at the beginning of the bonding procedure for aligner treatments. Both insufficient and excessive adhesives can lead to underfilled or overcontoured attachment surfaces after light curing, which can compromise force transmission during aligner treatment. In this context, the present study is the first study to evaluate the impact of using a dental loupe with 2.5X magnification during the bonding phase on both attachment accuracy and the amount of flash adhesive. The present findings show that the null hypothesis was mostly rejected. This investigation led to the conclusion that the use of dental magnifying loupes during the placement of adhesives into the reservoirs could improve the accuracy of attachment and reduce the amount of flash adhesive.

In the orthodontic literature, many studies have examined the effectiveness of using dental loupes during debonding phase. Alencar et al.^19^ assessed the effectiveness of visual magnification in removing composite flash around metal brackets. They concluded that the use of a light-headed magnifying glass offered superior performance compared to removal performed with the naked eye. In the study conducted by Ghaleb et al.^20^, it was concluded that the use of a magnifying loupe significantly facilitated the removal of adhesive resin -regardless of the system used for resin removal and finishing- by minimizing enamel damage and surface roughness, while also reducing the time required for the procedure. Similarly to the results of Ghaleb et al.,^20^ Baumann et al.^18^ also showed the decreased amount of enamel damage, line angle grooves, and composite residues on all teeth using 2.5X dental loupes.

Considering the aforementioned studies and the existing literature, there is, unfortunately, a limited number of studies evaluating the effectiveness of loupe use during orthodontic bonding procedure. Minimizing errors in the bonding process necessitates a high level of care and attention. Only one study^21^ has evaluated the impact of using loupes on bracket placement accuracy in fixed orthodontic appliances. The findings revealed no statistically significant difference in the error for bracket positioning with and without loupes, suggesting that magnification did not offer a notable advantage over visual assessment with the naked eye. 

The present study found no significant difference between groups in the mean “defect” measurement of placement accuracy. This indicates that 2.5× magnification did not enhance the operator’s ability to prevent underfilled areas during the bonding process. However, in contrast to this finding, the median “excess” measurement accuracy of attachments in Group 1 (with a loupe) (0.38 mm) was significantly lower than that of Group 2 (without a loupe) (0.46 mm). This result emphasized that using a magnifying loupe during the bonding of aligner attachments potentially affected the precision of attachment shape reproduction. Although previous research^21^ has shown no significant benefit of loupes for bracket placement accuracy, attachment bonding requires a higher level of visual precision. Attachments are smaller, lack clear geometric boundaries, and are highly sensitive to overfill or underfill of composite material, making magnification more relevant. In addition, template-guided placement and the translucency of the composite increase the need to evaluate fine detail, whereas bracket placement relies on well-defined reference points and is less affected by minor deviations. These factors may explain why magnification enhances attachment accuracy but not bracket positioning. Since all aligners are fabricated according to the initial attachment template, any inaccuracies occurring during the initial bonding phase may result in improper tooth movements that cannot be corrected by subsequent aligners throughout the treatment process.

When considering the flash adhesive area of the composite that occurs around the attachment during each bonding process, the “without loupe” group showed a statistically significantly higher value (42.01±10.32 mm^2^) compared to the “with loupe” group (31.79±5.38 mm^2^) (p<0.001). As noted in a study^24^, composite materials undergo shade changes over time, potentially compromising aesthetic outcomes. Moreover, adhesive remnant areas can form raised and rougher surfaces that facilitate biofilm accumulation and increase the risk of caries development.^3^ ^,^ ^4^ ^,^ ^25^ ^-^ ^27^ The findings of the current study highlight that accurate bonding of attachments can be achieved by avoiding excessive overfilling of the template reservoirs, thereby minimizing flash formation with the use of dental loupes. This approach may enable more precise tooth movements and improve the predictability of treatment outcomes, while also reducing the risk of iatrogenic damage and cross-contamination from aerosols produced by rotary instruments.^28^ Moreover, this method (bonding aligner attachment with a dental loupe) may reduce the need for additional instrumentation and contribute to a more time-efficient clinical workflow. A systematic review evaluating interventions for musculoskeletal symptoms found that the use of equipment -such as magnification loupes in combination with indirect vision- significantly reduces musculoskeletal issues in both the upper and lower body.^29^ On the other hand, dental loupes present certain drawbacks that warrant consideration. A key limitation to their widespread use in Dentistry is their limited accessibility, as the acquisition and maintenance of high-quality loupes involve significant financial investment. As highlighted in a systematic review^22^, greater efforts are needed to encourage the early adoption of magnification loupes during the formative stages of dental professionals’ careers.

Several limitations of this study should be acknowledged. Primarily, it was conducted *in vitro*, which does not fully replicate the intraoral environment. In addition, the relatively small sample size may affect the generalizability of the findings. The outcomes of the bonding tests presented in this study may be influenced by both the operator performing the procedures and the mechanical characteristics of the aligner material employed. Although the experiments were performed by an experienced aligner practitioner following clinical guidelines,^30^ future studies may expand the methodology by evaluating different attachment designs, lower-viscosity flowable resins, and simulated posterior access under limited visibility.

## CONCLUSIONS

Using a dental loupe for magnification improved the accuracy of aligner attachment bonding procedures and decreased the adhesive flash area around the attachments.

These findings support that magnifying loupes may serve as a useful adjunct for enhancing procedural precision during aligner attachment bonding in orthodontic practice.

## Data Availability

Due to the sensitive nature of the data and ethical restrictions, the datasets supporting this study are not publicly available.
